# Genetically predicted vitamin C levels significantly affect patient survival and immunotypes in multiple cancer types

**DOI:** 10.3389/fimmu.2023.1177580

**Published:** 2023-05-22

**Authors:** Jing Yuan, Yu-hong Zhang, Xin Hua, Hui-qi Hong, Wei Shi, Kun-xiang Liu, Ze-xian Liu, Peng Huang

**Affiliations:** ^1^ Sun Yat-Sen University Cancer Center, State Key Laboratory of Oncology in South China, Collaborative Innovation Center for Cancer Medicine, Guangzhou, China; ^2^ Department of Radiation Oncology, Ruijin Hospital, Shanghai Jiaotong University School of Medicine, Shanghai, China; ^3^ Department of Oncology, Shunde Hospital of Southern Medical University, Foshan, China; ^4^ State Key Laboratory of Applied Optics, Changchun Institute of Optics, Fine Mechanics and Physics, Chinese Academy of Sciences, Changchun, China; ^5^ University of Chinese Academy of Sciences, Beijing, China; ^6^ Metabolomics Innovation Center, Sun Yat-sen University Zhongshan School of Medicine, Guangzhou, China

**Keywords:** vitamin C, pan-cancer analysis, prediction model, prognosis, immunotypes

## Abstract

**Background:**

Recent observational studies and meta-analyses have shown that vitamin C reduces cancer incidence and mortality, but the underlying mechanisms remain unclear. We conducted a comprehensive pan-cancer analysis and biological validation in clinical samples and animal tumor xenografts to understand its prognostic value and association with immune characteristics in various cancers.

**Methods:**

We used the Cancer Genome Atlas gene expression data involving 5769 patients and 20 cancer types. Vitamin C index (VCI) was calculated using the expression of 11 genes known to genetically predict vitamin C levels, which were classified into high and low subgroups. The correlation between VCI and patient overall survival (OS), tumor mutational burden (TMB), microsatellite instability (MSI), and immune microenvironment was evaluated, using Kaplan-Meier analysis method and ESTIMATE (https://bioinformatics.mdanderson.org/estimate/). Clinical samples of breast cancer and normal tissues were used to validate the expression of VCI-related genes, and animal experiments were conducted to test the impact of vitamin C on colon cancer growth and immune cell infiltration.

**Results:**

Significant changes in expression of VCI-predicted genes were observed in multiple cancer types, especially in breast cancer. There was a correlation of VCI with prognosis in all samples (adjusted hazard ratio [AHR] = 0.87; 95% confidence interval [CI] = 0.78–0.98; *P* = 0.02). The specific cancer types that exhibited significant correlation between VCI and OS included breast cancer (AHR = 0.14; 95% CI = 0.05–0.40; *P* < 0.01), head and neck squamous cell carcinoma (AHR = 0.20; 95% CI = 0.07–0.59; *P* < 0.01), kidney clear cell carcinoma (AHR = 0.66; 95% CI = 0.48–0.92; *P* = 0.01), and rectum adenocarcinoma (AHR = 0.01; 95% CI = 0.001–0.38; *P* = 0.02). Interestingly, VCI was correlated with altered immunotypes and associated with TMB and MSI negatively in colon and rectal adenocarcinoma (*P* < 0.001) but positively in lung squamous cell carcinoma (*P* < 0.05). *In vivo* study using mice bearing colon cancer xenografts demonstrated that vitamin C could inhibit tumor growth with significant impact on immune cell infiltration.

**Conclusion:**

VCI is significantly correlated with OS and immunotypes in multiple cancers, and vitamin C might have therapeutic potential in colon cancer.

## 1 Introduction

Vitamin C is an essential element involved in many human physiological activities. This compound plays a major role in collagen synthesis, oxygen sensing, and regulation of epigenetics and host immunity (1–7). Several meta-analyses have demonstrated that higher vitamin C levels/intake are associated with reduced cancer incidence and mortality (8–16). Vitamin C is also an electron donor that affects extracellular matrix remodeling and could affect cancer metastasis (17), which is a major cause of poor prognosis in multiple cancer types (18, 19). Vitamin C has preclinical and clinical therapeutic effects on cancer (20, 21). Recent preclinical studies have suggested potential benefit of vitamin C in cancer immunotherapy (4, 22), but whether Vitamin C has specific correlations with tumor immunological features remains unclear.

Although many observational and experimental studies support an inverse relationship between vitamin C levels and cancer (23–25), randomized controlled trials examining the therapeutic effect of vitamin C supplementation in cancer patients have reported inconsistent results (21, 26, 27). Moreover, increased Vitamin C consumption due to cancer-related oxidative stress activity and decreased dietary vitamin C supplementation due to cancer-generated symptoms (dysphagia, vomiting, nausea, abnormal taste) may contribute to its pseudo-prognostic cancer results. Therefore, the relationship between vitamin C and cancer prognosis and the positive or negative directions of this correlation, if it exists, require further exploration. Both data analysis using pan-cancer datasets in public databases and specific laboratory experiments are necessary to provide further insights in this area.

Mendelian randomization studies have used genetically predicted Vitamin C levels to explore the relationship between Vitamin C and cardiovascular disease, type 2 diabetes, and cancer risk (28–30). Based on vitamin C predictive measures generated by Mendelian randomization analysis, we performed a pan-cancer analysis for the first time to examine whether intrinsically predicted vitamin C levels could influence cancer prognosis. Laboratory study was performed to further valid these findings.

## 2 Materials and methods

### 2.1 Analysis of public database and cancer types

RNA sequencing (HTSeq-fragments per kilobase per million [FPKM]), clinicopathological, and survival data from patients with >30 types of cancers from The Cancer Genome Atlas (TCGA) database were downloaded from the UCSC Xena database (https://xena.ucsc.edu/) (31). Since vitamin C has a reinforcement effect in immunotherapy (4, 24) and tumor mutational burden (TMB) and microsatellite instability (MSI) could predict cancer immunotherapy response (32, 33), we evaluated cancer immunological features using TMB, MSI, and immunotype correlation analyses (34–37). The TMB value can be calculated using perl language or the maftools package of R software, and the MSI score of each sample can be calculated using MANTIS algorithm. The correlation between the VCI and TMB or MSI was analyzed using the R language fmsb package (https://cran.r-project.org/web/packages/fmsb/index.html). Tumor immune microenvironment (TME) scores and tumor immune cell infiltration were analyzed using the R language ESTIMATE and CIBERSORT algorithms, respectively. Patient overall survival (OS) was used as the key indicator of prognostic outcome, as it is the universal gold-standard prognosis in clinical practice (38).

The pan-cancer analysis included samples from 20 cancer types (n = 5769) with clinicopathological (age, gender, race, disease stage) and survival information. This includes bladder urothelial carcinoma (BLCA, n = 385), breast invasive carcinoma (BRCA, n = 973), cholangiocarcinoma (CHOL, n = 36), colon adenocarcinoma (COAD, n = 269), esophageal carcinoma (ESCA, n = 124), head and neck squamous cell carcinoma (HNSC, n = 419), kidney chromophobe (KICH, n = 62), kidney renal clear cell carcinoma (KIRC, n = 517), kidney renal papillary cell carcinoma (KIRP, n = 245), liver hepatocellular carcinoma (LIHC, n = 334), lung adenocarcinoma (LUAD, n = 440), lung squamous cell carcinoma (LUSC, n = 382), mesothelioma (MESO, n = 84), pancreatic adenocarcinoma (PAAD, n = 170), rectum adenocarcinoma (READ, n = 78), skin cutaneous melanoma (SKCM, n = 415), stomach adenocarcinoma (STAD, n = 291), testicular germ cell tumors (TGCT, n = 78), thyroid carcinoma (THCA, n = 413), and uveal melanoma (UVM, n = 54). Our screening criteria required that all cancer patients in the database must have all information on age, gender, race, and pathological stage indicators for multi-factor analysis. Patients with incomplete information or whose survival follow-up time less than 30 days were excluded. The baseline patient characteristics of the 20 cancer types included in our data analysis are shown in **Supplementary Table S1**.

### 2.2 Genetically predicted vitamin C levels

Based on previous population-based Mendelian randomization analysis, the tumor genetically predicted vitamin C levels were determined based on the expression of 11 relevant genes obtained from genome-wide association study (28). For quantitative characterization of the genetically predicted citamin C levels across different cancers, we calculated the vitamin C index using the following formula: VCI = 0.04 × exp*AKT1 + *0.063 × exp*BCAS3 + *0.058 × exp*CHPT1 + *0.036 × exp*FADS1 + *0.038 × exp*GSTA5 + *0.041 × exp*MAF* + 0.039 × exp*RER1 + *0.039 × exp*RGS14 + *0.36 × exp*SLC23A1 + *0.102 × exp*SLC23A3 + *0.078 × exp*SNRPF.* The VCI values of all samples were classified into high and low subgroups, using the optimal cutoff value of 1.50 determined by the maximally selected rank statistics for OS as described previously (39).

### 2.3 Gene set enrichment analysis (GSEA)

Gene set enrichment analysis (https://www.gsea-msigdb.org/gsea/index.jsp) was conducted on high vs. low VCI subgroups to determine the pathway and biological function differences (40). We used the web tools c2.cp.kegg.v7.2.symbols.gmt and h.all.v7.2.symbols.gmt in Molecular Signatures Database (MSigDB) for our analyses. Each GSEA process was repeated 1000 times, and gene sets with *P* < 0.05 and false discovery rate < 0.25 were considered significantly enriched.

### 2.4 Tumor xenograft and animal study

Animal study using mice bearing colon cancer xenografts was approved by the Animal Care and Use Committee of sun Yat-sen University Cancer Center. Female BALB/c mice (age 8-9 weeks, 12 mice) were purchased from Beijing Victoria River Experimental Animal Technology Co. LTD. (Beijing, China). All mice were fully randomized before the experiment and were given a concentrated and regular dose of sterilized feed and sterile drinking water. To test the effect of vitamin C on tumor growth in mice, CT26 cancer cells were injected subcutaneously into the right side of BALB/C mice. After 7 days of tumor cell inoculation, vitamin C was injected intraperitoneally once every 2 days (500mg/kg). By the end of the experiments (3 weeks), the mice were sacrificed and tumor tissues were isolated, weighted, and sliced for analysis of immune cell infiltration using flow cytometry and immunohistochemistry.

### 2.5 Immunohistochemistry (IHC)

The immunohistochemical process is performed as follows. Briefly, tumor tissue slices were first dried at 58°C for 1 hour and then dewaxed by heating at 100 °C for 5 minutes in 10 mM sodium citrate (pH6.0). In order to eliminate the activities of endogenous peroxidase and alkaline phosphatase in the tissues, the tissue sections were treated with 3% hydrogen peroxide for 15 min, then incubated with the indicated specific antibody overnight, followed by washing and incubation with secondary antibody for 1.5 h. DAB was used as the color substrate for revelation of the antigen. The slices were countered stained with hematoxylin.

### 2.6 Flow cytometry analysis of immune cell infiltration

The tumor tissues were digested into single cells using collagenase III (200U/mL) and DNase (50U/mL). The immune cells in tumor tissue suspension were stained with the respective antibodies and analyzed using the Beckman Coulter flow cytometry with CytExpert software (Miami, FL, USA).

### 2.7 Real-time RT-PCR

Total RNA was isolated from tumor tissues Trizol Reagent (Invitrogen Life Technologies) according to manufacturer’s instructions. Primer Script RT reagent Kit with gDNA Eraser (Takara BIO INC, Kusatsu, Shiga,Japan) was used for reverse transcription of RNA. Real-time quantitative PCR was performed using the SYBR Premix Ex Taq RNAse H+ kit (Takara), and the results were analyzed using the Bio-RAD detection system (Bio-RAD, USA).

### 2.8 Statistical analysis

Continuous data are reported as medians with interquartile ranges (IQR); categorical data are reported as frequencies with percentages, and compared using the Mann–Whitney U test, chi-square test, continuity corrected chi-square test, or Fisher’s exact test where appropriate. The cancer patient OS was estimated using the Kaplan–Meier method and compared by the log-rank test. The Cox proportional hazards model was performed to calculate the adjusted hazard ratios (HRs) and the corresponding 95% confidence intervals (CIs), adjusted with age, gender, race, and disease stage. All statistical analyses were conducted using R version 4.0.2 (http://www.r-project.org). Statistical significance was set at two‐sided *P* < 0.05. For laboratory experiments, all lab tests were performed at least 3 times (3 independent replicates). The Student t test was used to evaluate the statistical significance of the difference between two groups. Statistical analysis was performed using GraphPad Prism 8.0 (San Diego, CA, USA) and SPSS 20.0 (Chicago, IL, USA) software.

## 3 Results

### 3.1 Vitamin C index in cancer and normal tissues

**Figure 1:**
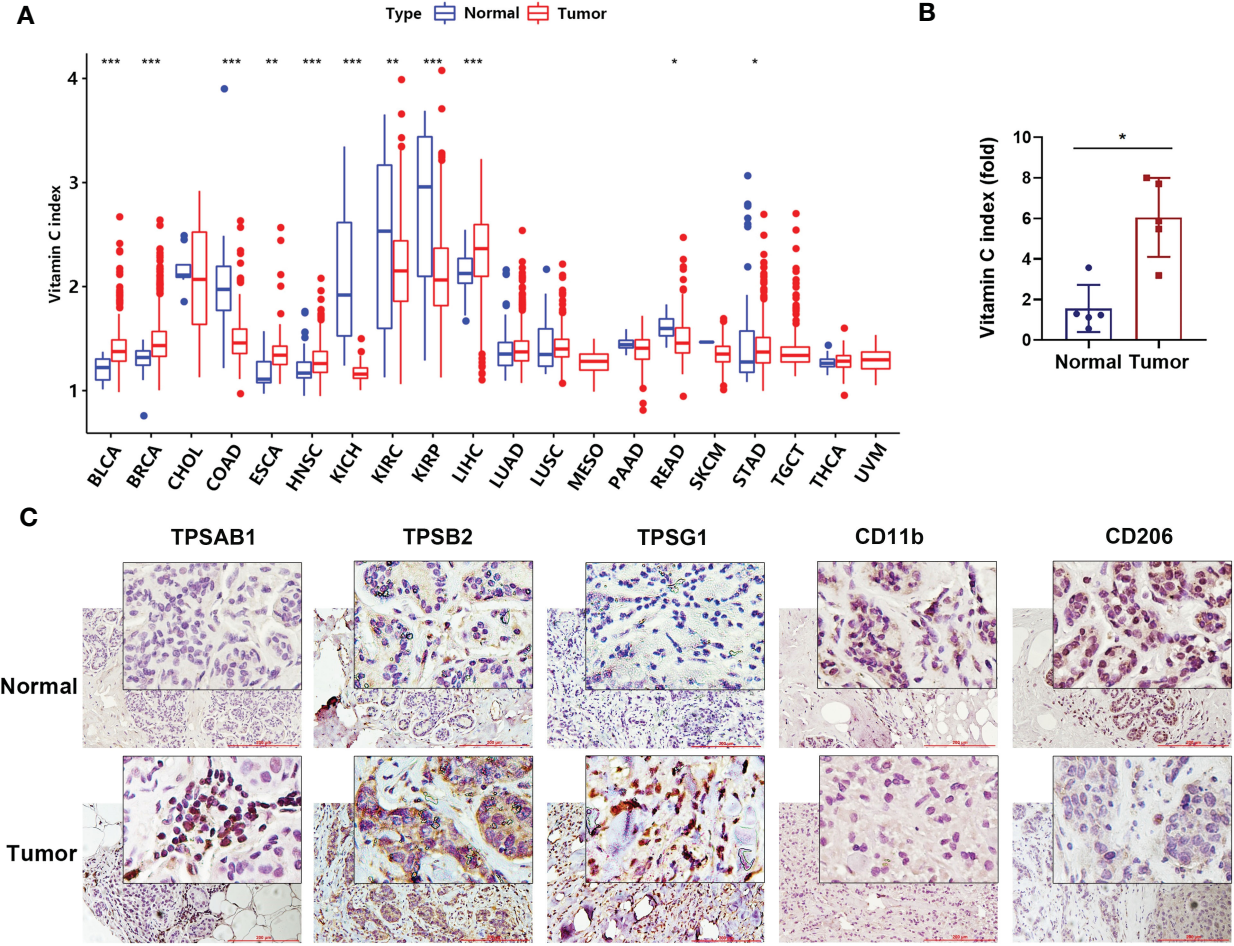
Genetically predicted vitamin C index in human tumor and normal tissues. **(A)** Comparison of vitamin C index (VCI) in 20 types of tumor issues (indicated by red color) and their respective normal tissues (blue color). VCI values were calculated be the expression of the 11 vitamin C-predictive genes as described under Methods. The names of cancer types are indicated below the horizonal line. **(B)** Vitamin C index in human breast cancer tissue and the adjacent normal tissue (n=5 pairs). The mRNA levels of the expression of the 11 vitamin C-predictive genes were quantified by qRT-PCR for calculation of VCI. **(C)** Representative images of breast cancer and normal tissues immune-stained with Tryptase alpha/beta1 (TPSAB1), Tryptase beta2 (TPSB2), Tryptase gamma1 (TPSG1), CD11b, and CD206 antibodies as indicated. Date are means ± SD of three separate experiments; *P < 0.05; **P < 0.01; ***P < 0.001.

Using the TCGA datasets, we analyzed vitamin C index (VCI) of 5769 patients from 20 cancer types based on the expression of 11 genes known to predict vitamin C levels. **Table S1** summarizes baseline patient characteristics: 2973 patients (52%) were male and 2796 (48%) were female. The median age was 61 years (IQR, 51–71); the median OS was 2.0 years (IQR, 1.1–3.9). We first compared VCI values in the 20 types of cancer tissues and their respective normal tissues, and found a significant difference between them in the majority of cancer types, including BLCA, BRCA, COAD, ESCA, HNSC, KICH, KIRC, KIRP, LIHC, READ, and STAD (**Figure 1A**). For instance, analysis of the breast cancer datasets revealed that VCI was significantly higher in breast cancer tissues compared to the normal breast tissues. We then performed experiments to validated this finding by analyzing the mRNA expression of the 11 VCI-predicting genes in breast cancer tissues in comparison with the adjacent normal breast tissues, and found that the VCI value in breast cancer was more than 3-fold higher than that in the normal tissues (**Figure 1B**). Interestingly, immunohistochemical staining of the breast tissues showed that the expressions of CD11b (a pan-macrophage marker) and CD206 (a M2-like macrophage marker) were both low while the expression of Tryptase alpha/beta1, beta2 and gamma (all mast cell marker) was high in the breast cancer tissues (**Figure 1C**). Since CD206 is involved in antigen capture in humoral immune response and plays a role in antigen transport, whereas CD11b is known to mediate macrophage adhesion, migration, chemotaxis and accumulation, the presence of CD11b^+^/CD206^+^ tumor-associated macrophages could promote the activation of specific CD8+ T cells associated with improved overall survival of patients (41). Thus, a reduction of CD11b^+^ cells would suggest a polarization of immunosuppressive macrophages in favor of tumor growth. These results suggest that the level of vitamin C could affect the infiltration of certain immune cells, and prompted us to analyze the relationship between VCI and immunotypes in various cancer types (see Section 3.3).

Based on the distribution of VCI and the maximally selected rank statistics, the patients were stratified into high-VCI and low-VCI subgroups with a cutoff value of 1.5 (**Figure S1**). There were 3751 patients (65%) and 2018 patients (35%) in the low-VCI and high-VCI groups, respectively. The patient clinicopathological characteristics including age, gender, race, and disease stages were compared between the two VCI subgroups (**Table S2**). We found a significant difference between the two VCI subgroups in their clinicopathological characteristics. Younger patients had higher VCI than older patients, especially in BRCA, BLCA and HNSC cancers; male patients had higher VCI than female patients, especially in HNSC, KIRC and STAD cancers; patients in the high-VCI also tended to have lower disease stage, whereas low-VCI patients were more likely in the advanced stage.

### 3.2 Impact of vitamin C index on cancer patient survival

**Figure 2:**
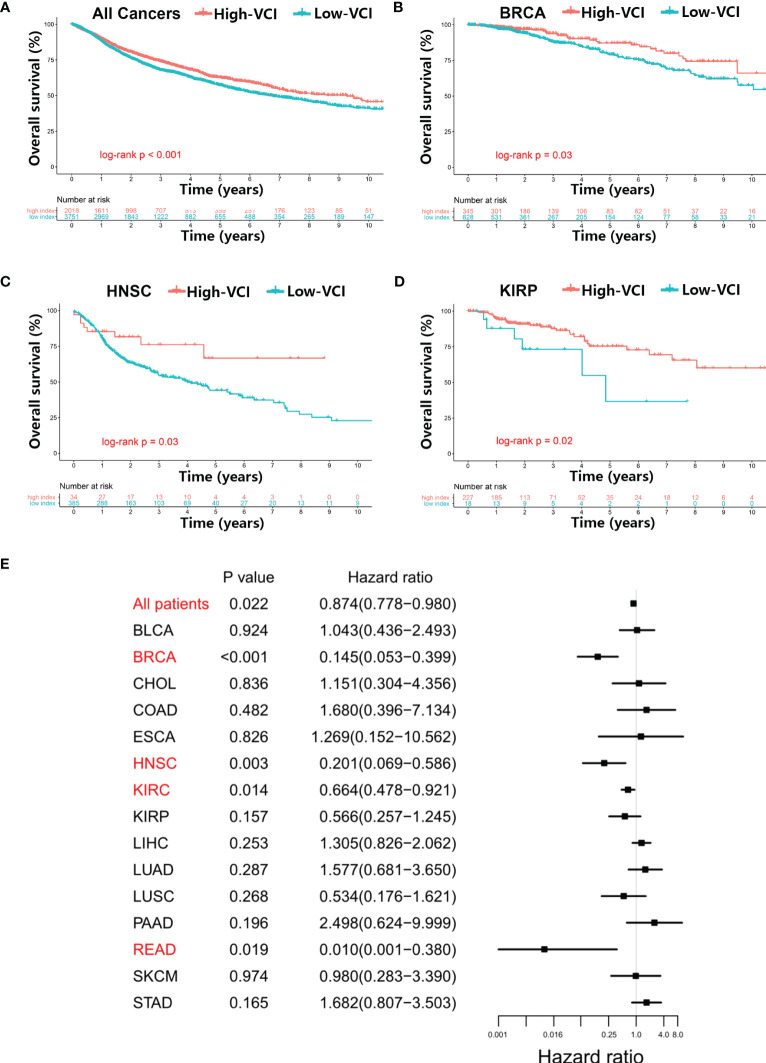
Relationship between vitamin C index and overall survival of cancer patients. **(A)** Kaplan–Meier survival analysis in all 5769 patients of 20 cancer types with high or low vitamin C index (VCI), and in specific cancer types including breast cancer **(B)**, head and neck squamous cell carcinoma **(C)**, and kidney renal papillary cell carcinoma **(D)**, using datasets from TCGA database. **(E)** Cox proportional hazards analysis of VCI across multiple cancer types, using variates including age (continuous variable), gender (male vs. female), race (white vs. Asian vs. other), and disease stage (stage III–IV vs. stage I–II) as clinicopathological variates for adjustment in the multivariable model analysis.

As the values of VCI varied substantially in different cancer types and among patients within a same cancer type (**Figure 1A**), we tested whether VCI might have any impact on cancer patient survival. Kaplan–Meier survival analysis was used to evaluate the overall survival (OS) of high-VCI cancer patients in comparison with low-VCI patients. Overall, among all 5769 patients of 20 cancer types, patients with high-VCI exhibited significantly better OS than those with low-VCI (**Figure 2A**, *P* < 0.01). This observation prompted us to further analyze the impact of VCI on OS within a particular cancer type. We found that the high-VCI group had significantly better survival than the low-VCI group in breast cancer (**Figure 2B**, *P* = 0.03), kidney renal papillary cell carcinoma (**Figure 2C**, *P* = 0.02), and head and neck squamous cell carcinoma (**Figure 2D**, *P* = 0.03). There was marginal significance for lung squamous cell carcinoma (**Figure S2A**, *P* = 0.08). The VCI did not show significant impact on patient survival in other cancer types (**Figures S2B–L**). Four cancer types (KICH, MESO, THCA, UVM) were not included in this comparison analysis since KICH patients were all in the low-VCI group whereas MESO, THCA and UVM patients were all in the high-VCI group. Testicular germ cell tumor was not included in this survival comparison analysis because of limited patient number with insufficient OS endpoint events.

We then tested whether the vitamin C index could independently influence cancer prognosis, using multivariate Cox analysis that incorporated clinicopathological covariates (age, gender, race, disease stage) for adjustment (**Figure 2E**, **Table S3**). The multivariable modeling results were generally consistent with the findings from Kaplan‐Meier univariate analysis. The vitamin C index independently influenced the overall survival when all 5769 patients were included in the analysis (adjusted hazard ratio [AHR] = 0.87; 95% CI = 0.78–0.98; *P* = 0.02). Among the specific cancer types, breast cancer (AHR = 0.14; 95% CI = 0.05–0.40; *P* < 0.01), head and neck squamous cell carcinoma (AHR = 0.20; 95% CI = 0.07–0.59; *P* < 0.01), kidney renal papillary cell carcinoma (AHR = 0.66; 95% CI = 0.48–0.92; *P* = 0.01), and rectum adenocarcinoma (AHR = 0.01; 95% CI = 0.001–0.38; *P* = 0.02) exhibited statistical significance (**Figure 2E**).

### 3.3 Relationship between vitamin C index and immunological features in cancer

**Figure 3:**
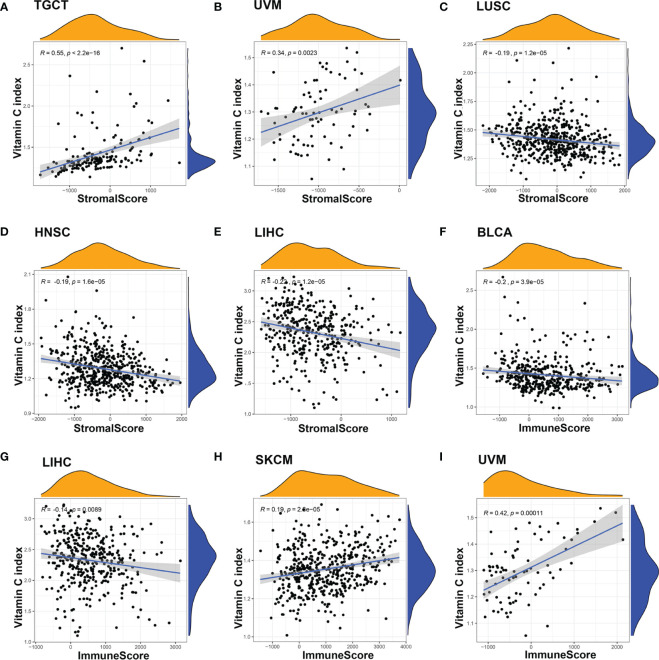
Correlation between Vitamin C index and the tumor immune microenvironment (TME). The correlation between VCI and TME were evaluated by calculating stromal scores and immune scores based on gene expression characteristics of stromal and immune cells using the ESTIMATE prediction software. There was a significant correlation between VCI and stromal score when all 20 cancer types were included in the analysis **(A)**. For specific cancer types, significant correlation was observed in UVM **(B)**, LUSC **(C)**, HNSC **(D)**, and LIHC **(E)**. The correlation between VCI and immune scores in BLCA **(F)**, LIHC **(G)**, SKCM **(H)**, and UVM **(I)** was also significant.

Based on the observation that normal and cancer tissues with different levels of VCI exhibited different infiltration of immune cells (**Figure 1C**), we further to analyzed the potential correlation between vitamin C index and tumor immunological features such as stromal scores and immune cell infiltrations, using the web tool ESTIMATE that calculates the scores of stromal cells (StromalScore) and the infiltration of immune cells (ImmuneScore) in the tumor tissues based on gene expression data (https://bioinformatics.mdanderson.org/estimate/). We found that VCI was significantly correlated with the ImmuneScore in multiple cancer types including BLCA (P < 0.001), LIHC (P < 0.001), LUAD (P < 0.05), SKCM (P < 0.001), STAD (*P* < 0.05), and UVM (*P* < 0.001) (**Figure 3**, **Figure S3**). VCI was also correlated significantly with the StromalScore in BLCA (*P* < 0.001), HNSC (*P* < 0.001), KIRC (*P* < 0.001), LIHC (*P* < 0.001), LUSC (*P* < 0.001), PAAD (*P* < 0.05), TGCT (*P* < 0.001), and UVM (*P* < 0.001) (**Figure 3**, **Figure S3**). Based on the above results, we used immunohistochemical method to detect the clinical breast cancer tissue and normal tissue fibroblasts markers VIMENTIN |, alpha SMA and endothelial cell marker CD31 expression. Results show that compared with normal tissue, the expression of VIMENTIN, α-SMA and CD31 in breast cancer tissues were significantly increased (**Figure S3F**).

**Figure 4:**
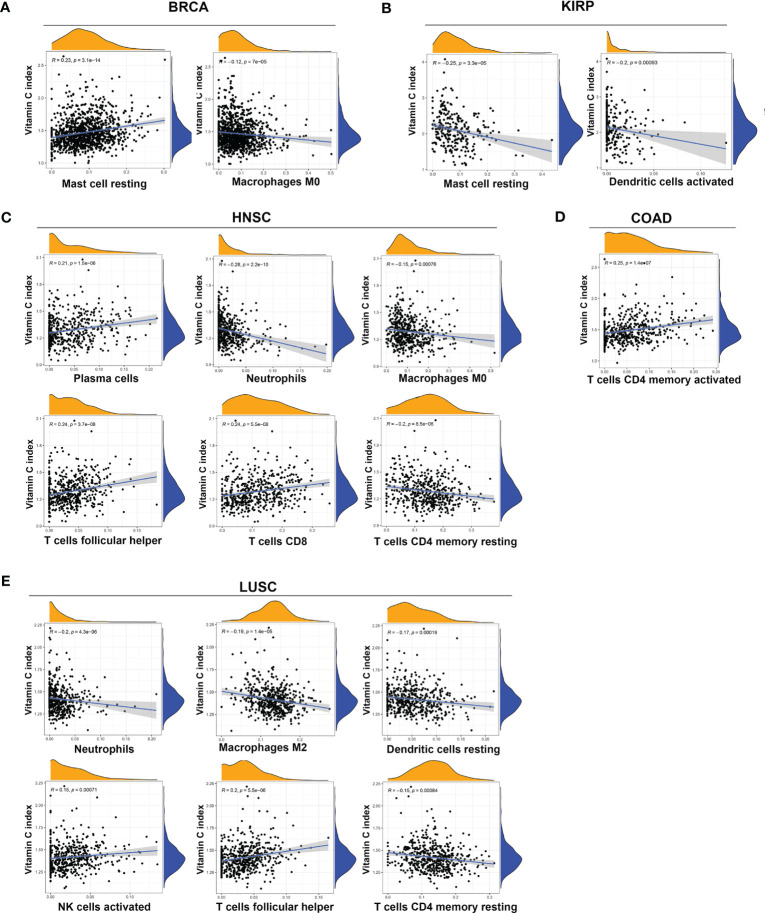
Association between vitamin C index and infiltration of immune cells in various cancers. Significant correlation between VCI and infiltration of immune cells (p < 0.001) was observed in BRCA **(A)**, KIRP **(B)**, HNSC **(C)**, COAD **(D)**, and LUSC **(E)**. The specific types of infiltrated immune cells are indicated at the bottom of each panel.

The association between the VCI and the infiltration of 22 types of specific immune cells was also analyzed using the CIBERSORT web tool (https://rdrr.io/github/IOBR/IOBR/man/CIBERSORT.html). The following significant associations, with P < 0.001, were found: BLCA was associated with infiltration of neutrophils and regulatory T cells (Tregs); BRCA was associated with infiltration of M0 macrophages and resting mast cells; COAD was associated with infiltration of memory activated CD4 T cells; HNSC was associated with infiltration of M0 macrophages, neutrophils, plasma cells, memory resting CD4 T cells, CD8 T cells, and follicular helper T cells; KIRC was associated with infiltration of resting natural killer cells; KIRP was associated with infiltration of activated dendritic cells and resting mast cells; LUAD was associated with infiltration of resting dendritic cells, M1 macrophages, and follicular helper T cells; LUSC was associated with infiltration of resting dendritic cells, M2 macrophages, neutrophils, activated NK cells, resting CD4 memory T cells, follicular helper T cells; SKCM was associated with infiltration of M2 macrophages, and activated CD4 memory T cells; STAD was associated with infiltration of plasma cells, and follicular helper T cells; TGCT (naïve B cells, M2 macrophages, activated CD4 memory T cells), THCA was associated with infiltration of memory B cells, activated dendritic cells, monocytes, resting CD4 memory T cells, CD8 T cells, and gamma delta T cells; UVM was associated with infiltration of M1 macrophages, monocytes, and follicular helper T cells (**Figure 4**, **Figure S4**). Taken together, VCI seemed to play complex roles in affecting stromal microenvironment and immune cell infiltration in a tissue-dependent manner.

### 3.4 Impact of vitamin C on tumor mutation burden, microsatellite instability, and tumor growth *in vivo*

**Figure 5:**
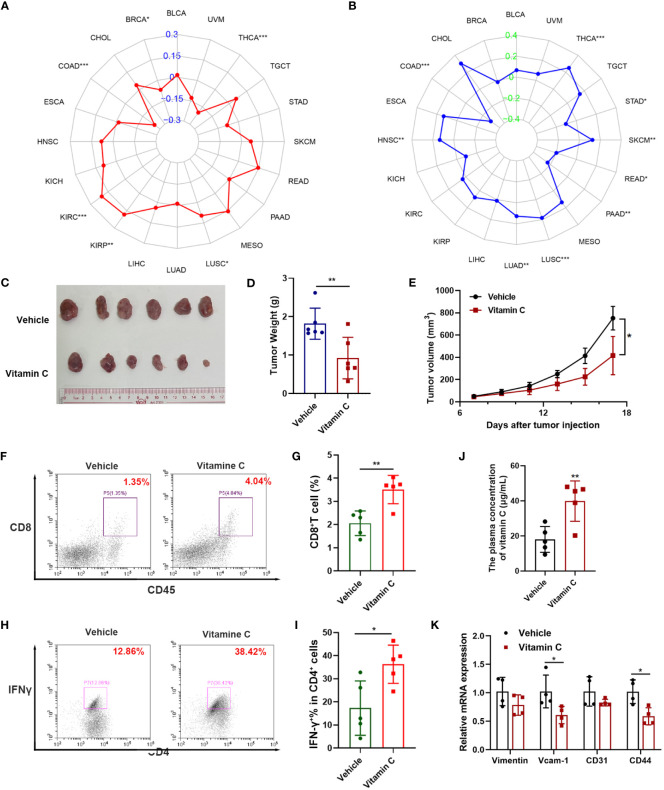
Impact of vitamin C on tumor mutation burden, microsatellite instability, and tumor growth *in vivo*. The relationship between the vitamin C index and tumor mutation burden **(A)**, and microsatellite instability **(B)** in various tumor types as indicated. **(C–E)** Balb/c mice inoculated with CT26 colon cancer cells (5 × 10^5) were treated with vitamin C (500mg/kg, i.p.) once every 2 days, and tumors were isolated, photographed, and weighted at the end of the experiment. The tumor volumes were measured every two days and tumor growth curves were shown, (mean ± SEM, n =6 per group). **(F, G)** Flow cytometry analysis of CD8^+^ T cells in CT26 tumor tissues. **(J)** Plasma concentrations of vitamin C in treatment and control groups. **(H)** Representative flow cytometric analysis of IFNγ^+^ CD4^+^ T cells in CT26 tumor tissues; **(I)** Quantitative data of IFNγ^+^ CD4^+^ T cells in CT26 tumor tissues. **(K)** The mRNA levels of VCAM-1, Vimentin, CD31 and CD44 in mouse tumor tissues were quantified by qRT-PCR *, P < 0.05; **, P < 0.01; ***, P < 0.001.

We also analyzed the potential correlation between VCI and tumor mutation burden (TMB) or microsatellite instability (MSI). As shown in **Figures 5A, B**, VCI exhibited a highly significant negative association with TMB and MSI in colon cancer (both, *P* < 0.001), and a significant positive association with TMB (*P* < 0.05) and MSI (*P* < 0.001) in LUSC. There was a significant negative association between VCI and TMB in BRCA (*P* < 0.05) and THCA (*P* < 0.001), and a significant positive association in KIRC (*P* < 0.001) and KIRP (*P* < 0.01); there was also a significant negative association between VCI and MSI in PAAD (*P* < 0.01) and STAD (*P* < 0.05), and a significant positive association in HNSC (*P* < 0.01), LUAD (*P* < 0.01), SKCM (*P* < 0.01), and THCA (*P* < 0.001).

Since VCI exhibited strong correlation with both TMB **(** **Figure 5A** **)** and MSI **(** **Figure 5B** **)** and promoted the activation of T memory cells in colon cancer **(** **Figure 4D** **)**, we conducted animal experiments to test the effect of vitamin C on colon cancer growth and its impact on immune cells in mouse model. Immune-competent Balb/C mice were inoculated subcutaneously with CT26 colon cancer cells, and treated vitamin C *via* intraperitoneal injections. The results showed that vitamin C was able to significantly inhibit the growth of CT26 tumor *in vivo* (**Figures 5C–E**). Flow cytometry analysis showed an increase in the proportion of CD8+ T cells in the tumor tissues from mice treated with vitamin C (**Figures 5F, G**). And vitamin C induced a major increase in IFN-γ-producing CD4+ cells in the tumor tissues (**Figures 5H, I**). In addition, plasma vitamin C concentration was significantly higher in the treatment group than in the control group (**Figure 5J**). Moreover, mRNA detection results of tumor tissues showed that vitamin C treatment significantly decreased the expression of VCAM-1, Vimentin, CD31 and CD44 (**Figure 5K**). These data together suggest that vitamin C was able to promote cancer immunity and might have promising therapeutic activity for treatment of colon cancer, which remains as a major challenge due to development of resistance to current therapeutic modalities (42, 43).

### 3.5 Gene set enrichment analysis based on vitamin C index

**Figure 6:**
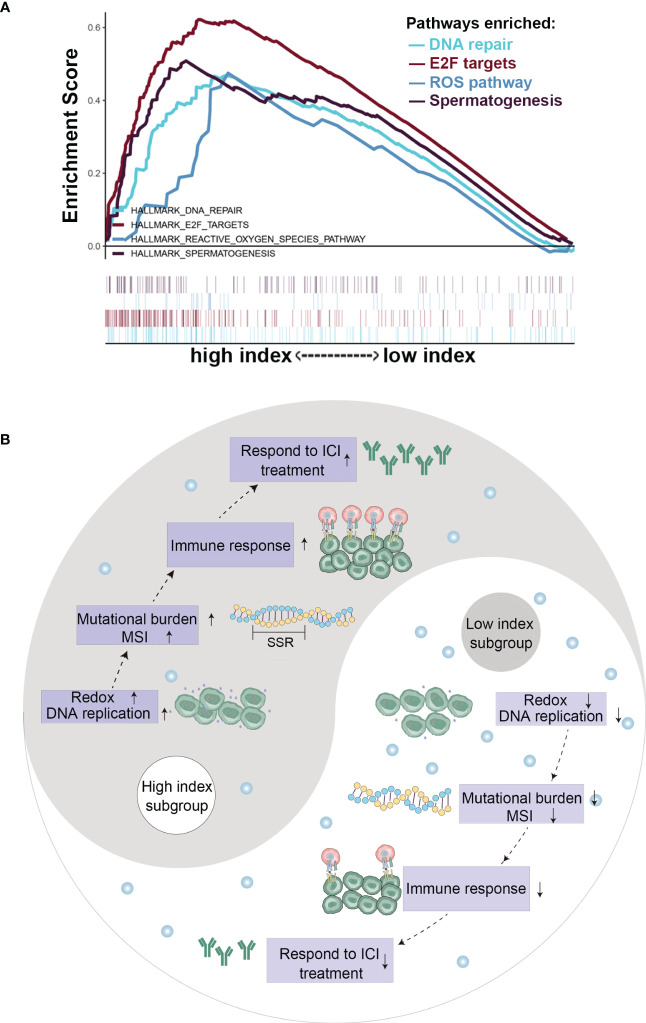
Gene expression and pathway analyses in cancer tissues with high or low vitamin C index. **(A)** Gene set enrichment analysis in head and neck cancer tissues with high or low vitamin C index; **(B)** Schematic illustration of the changes in key events and pathways in cancer with high VCI or low VCI.

To explore the potential molecular pathways by which vitamin C affects cancer growth and immune response, we conducted GSEA to determine the potential biological functions of the beneficial effect of gene set enrichment analysis (GSEA) in the high-VCI and low-VCI cancers. BRCA, HNSC, and KIRP were selected as representative cancers for this analysis, since VCI had significant impact on the patient survival and immunological features in these cancer types. Interestingly, the results of GSEA showed that VCI affected DNA damage repair, cell cycle-related pathway (E2F), and reactive oxygen species (ROS) pathway (**Figure 6A**). This seemed consistent with the findings that VCI was associated with tumor mutation burden and microsatellite instability (**Figures 5A, B**). Additional GSEA analyses showed that VCI was also associated with lipid and bile acid metabolism, and also with estrogen response (**Supplementary Figure S5**). These data together suggest that vitamin C could have complex impact on multiple pathways with a major effect on ROS and DNA stability, which could in turn regulate immune response since DNA fragments could activate the cGAS-STING pathway. **Figure 6B** illustrates the main pathways and biological processes that could be affected by VCI.

## 4 Discussion

In this pan-cancer study, we analyzed the expression of vitamin C-related genes from 5769 cancer patient samples in the TCGA database, and found a significant correlation between the genetically predicted Vitamin C levels and patient overall survival. Comprehensive analyses of immunological features showed a potential association between high-VCI and favorable response to immunotherapy. To the best of our knowledge, this was the first study that explored the potential prognostic value of VCI in multiple cancer types and investigated the vitamin effect on immunological features in various cancers. Laboratory study was also performed to validate some of the key findings form analyses of public datasets.

Several Mendelian randomization studies have previously explored the relationship between genetically predicted vitamin C levels and the risk of human diseases including diabetes, cardiovascular disease and cancer (27–29). A meta-analysis involving 45,758 patients demonstrated that each 50 μmol/L increase of vitamin C could lead to a 26% decrease in cancer risk (44). Observational studies have revealed that high-dose vitamin C exerts favorable antitumor effects in advanced cancers, even in chemotherapy-refractory patients (20). Since the level of vitamin C predicted by gene expression was significantly higher in breast cancer than in normal tissues (**Figure 1A**), we thus measured the expression of vitamin C-related genes in clinical samples of breast cancer patients compared to normal breast tissues, and the results were consistent with that found in the public database. In addition, we used the GCSA database, which contains TCGA cancer multi-omics data, to examine the copy number alterations/variations (CNV) of the Vitamin C gene family in pan-cancer. Additionally, the CNV percentage and the contribution of CNV to Vitamin C gene family expression were examined in each cancer. The heterozygous/homozygous CNV (deletion/amplification) status of the Vitamin C gene family exhibited a relatively comparable alteration trend, despite the Vitamin C gene showing diverse forms and amounts of CNV in different malignancies (**Figure S6**). We also found that high VCI was associated with better survival outcome in patients with BRCA, HNSC, and KIRP. Consistently, VCI seemed predictive of patient survival after adjustment for patient age and disease stage using multivariable models, indicating that vitamin C might independently influence prognosis through other biological functions rather than affecting tumor cell proliferation. One possibility is that vitamin C might promote immune functions and enhance immune cell infiltration in the tumor microenvironment, and thus exert its anticancer activity independent of direct effect on cancer cell. Interestingly, our analyses suggest that vitamin C was strongly correlated with both TMB (**Figure 5A**) and MSI (**Figure 5B**) in colorectal cancer. To experimentally test the effect of vitamin C on tumor growth and immune response *in vivo*, we selected a cancer type whose vitamin C level was relatively low so that the supplement of vitamin C would have a better chance to increase its level in the tumor tissue and thus modulate the immune cell functions to impact tumor growth. Based on the results of our analysis that colorectal cancer (COAD) exhibited a significantly lower vitamin C index (**Figure 1A**) correlated with alterations in T memory cells (**Figure 4D**), TMB (**Figure 5A**), and MSI (**Figure 5B**), we thus selected colon cancer model (CT26) for testing the therapeutic effect of vitamin C. The results indeed showed that the growth of CT26 xenografts was significantly inhibited by vitamin C treatment (**Figure 5C**), with improved infiltration of immune cells (**Figures 5E–H**), suggesting that vitamin C could have a potential in the treatment of colorectal cancer with low intrinsic vitamin C level.

The ability of vitamin C to promote immune functions has been known for a long time, and the underlying mechanisms are rather complex. Our study showed that VCI was significantly correlated with tumor mutation burden and microsatellite instability, and the gene set enrichment analysis revealed that VCI was associated ROS, DNA repair and cell cycle alterations, suggesting a possibility that DNA-damage response, which is known to activate the cGAS-STING, might be involved in the stimulation of immune response. As such, it would be interesting to consider the possibility to combine vitamin C with immunotherapy to improve the treatment outcome for cancer patients. Immune checkpoint inhibitors such as PD-l antibodies are important therapeutic agents that show promising anticancer activity against certain cancer. However, their efficacy differs significantly in different cancers and some cancer types such as pancreatic cancer and colon cancer exhibit low response rates to PD-1 antibody treatment. Thus, it would be worth of considering combination of vitamin C and immune checkpoint inhibitors for treatment of these cancer types. Our study showed that vitamin C index is positively correlated with the immune scores in lung adenocarcinoma, stomach adenocarcinoma, skin cutaneous melanoma, and uveal melanoma, and seemed to promote the infiltration of M1 macrophages, follicular helper T cells, activated NK cells and CD8+T cells in the tumor tissues. Thus, combination of vitamin C with immunotherapy could be tested in these cancer types. Interestingly, we found that vitamin C index was negatively correlated with the immune score in bladder urothelial carcinoma, which seemed to be associated with a reduction of neutrophils. Thus, the specific impact of vitamin C on immune response might be rather complex and likely cancer type-dependent. Obviously, future study in the laboratory and clinical settings are required in this area.

Beside its effect on immune functions, vitamin C can affect multiple biological processes, including inhibition of glucose transportation and ATP production (45), inducing DNA damage and promoting pro-oxidant effects (46). Our GSEA results are consistent with these multifaceted effects. The impact of vitamin C were enriched in gene sets associated with nutrient metabolism, oxygen sensing, peroxisome, and DNA repair pathways. Further studies are needed to evaluate the relative contributions of these pathways to the anticancer activity of vitamin C. It is possible that this might vary in different cancers due to their different genetic makeups and metabolic wirings. The definite correlation between Vitamin C and prognosis has facilitated pilot clinical trials for evaluating its beneficial effect in improving cancer survival outcomes and responses. Other study demonstrated that Vitamin C combined with hyperthermia improved survival in advanced, refractory non-small cell lung cancer (47). Nevertheless, the positive effect of vitamin C in promoting anticancer immunity and its negative impact against cancer cells *via* its pro-oxidant and DNA-damaging effect suggest that this compound may be useful as an anticancer agent. Considering vitamin C is a safe compound for use in human, its use as the drug in combination with immune checkpoint inhibitors and other anticancer agents for clinical treatment of cancer is highly feasible. In fact, a meta-analysis involving 23 single-arm and randomized phase I/II trials revealed the general safety and potential therapeutic effects of vitamin C (48). Based on the results of our study, it seems that many cancer types, especially BRCA, HNSC and KIRP may be appropriate for testing the therapeutic benefits of vitamin C, either alone or in combination with other drugs.

## 5 Conclusions

In summary, this study characterized the effect of vitamin C in 20 tumor types through pan-cancer analyses of public datasets and laboratory study using clinical samples and mice bearing tumor xenografts. Our results showed that genetically predicted vitamin C levels (or vitamin C index) are significantly corrected with the overall survival of patients in multiple cancer types, and exerted major influence on the tumor microenvironment, immune cells and stromal cells. Vitamin C exhibited significant *in vivo* therapeutic activity against colon cancer in mice, associated with an increase in immune cell infiltration in tumor tissues. Our findings suggest a strong feasibility of using vitamin C in combination with immunotherapy to improve the treatment outcome of cancer patients. However, the limitations of this study, which largely based on analyses of gene expression from public datasets for calculation of VCI, stromal and immune scores, should be noted. Further laboratory studies and clinical trials are needed to comprehensively evaluate the therapeutic activity of vitamin C, alone or in combination with immune checkpoint inhibitors or other drugs, to identify the most effective treatment approach.

## Data availability statement

The original contributions presented in the study are included in the article/**Supplementary Material**. Further inquiries can be directed to the corresponding authors.

## Ethics statement

The studies involving human participants were reviewed and approved by Ethics Committee of Sun Yat-sen University Cancer Center. Written informed consent for participation was not required for this study in accordance with the national legislation and the institutional requirements. The animal study was reviewed and approved by Animal Care and Use Committee of Sun Yat-sen University Cancer Center.

## Author contributions

JY and XH designed the study and performed experiments; JY, Y-hZ, XH, H-qH, WS, K-xL, Z-xL, and PH participated in data analysis and interpretation; JY, Y-hZ, and PH wrote the manuscript. All authors contributed to the article and approved the submitted version.
