# Supplementary material for: Genetically predicted vitamin C levels significantly affect patient survival and immunotypes in multiple cancer types
Source: Front Immunol. 2023 May 22;14:1177580. doi: 10.3389/fimmu.2023.1177580 (PMC10239825; doi:10.3389/fimmu.2023.1177580)
Supplement: Supplementary file 1 [file DataSheet_1.pdf]

Supplementary Figure S1

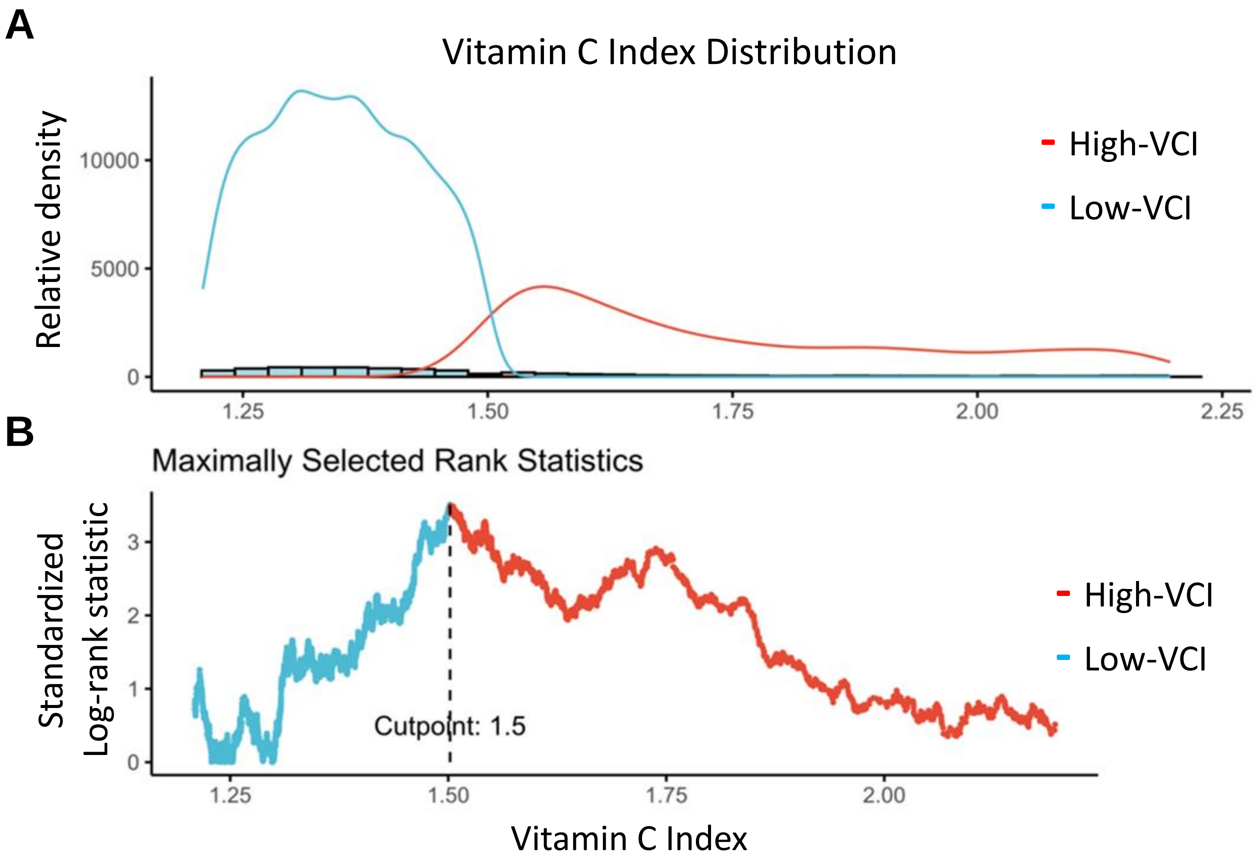

Fig S1. Determination of optimal cutoff value for vitamin C index. The vitamin C index (VCI) cutoff value of 1.50 was determined according to maximally selected rank statistics based on the VCI distribution (**A**) and standardized Log-rank statistical analysis (**B**).

Supplementary Figure S2

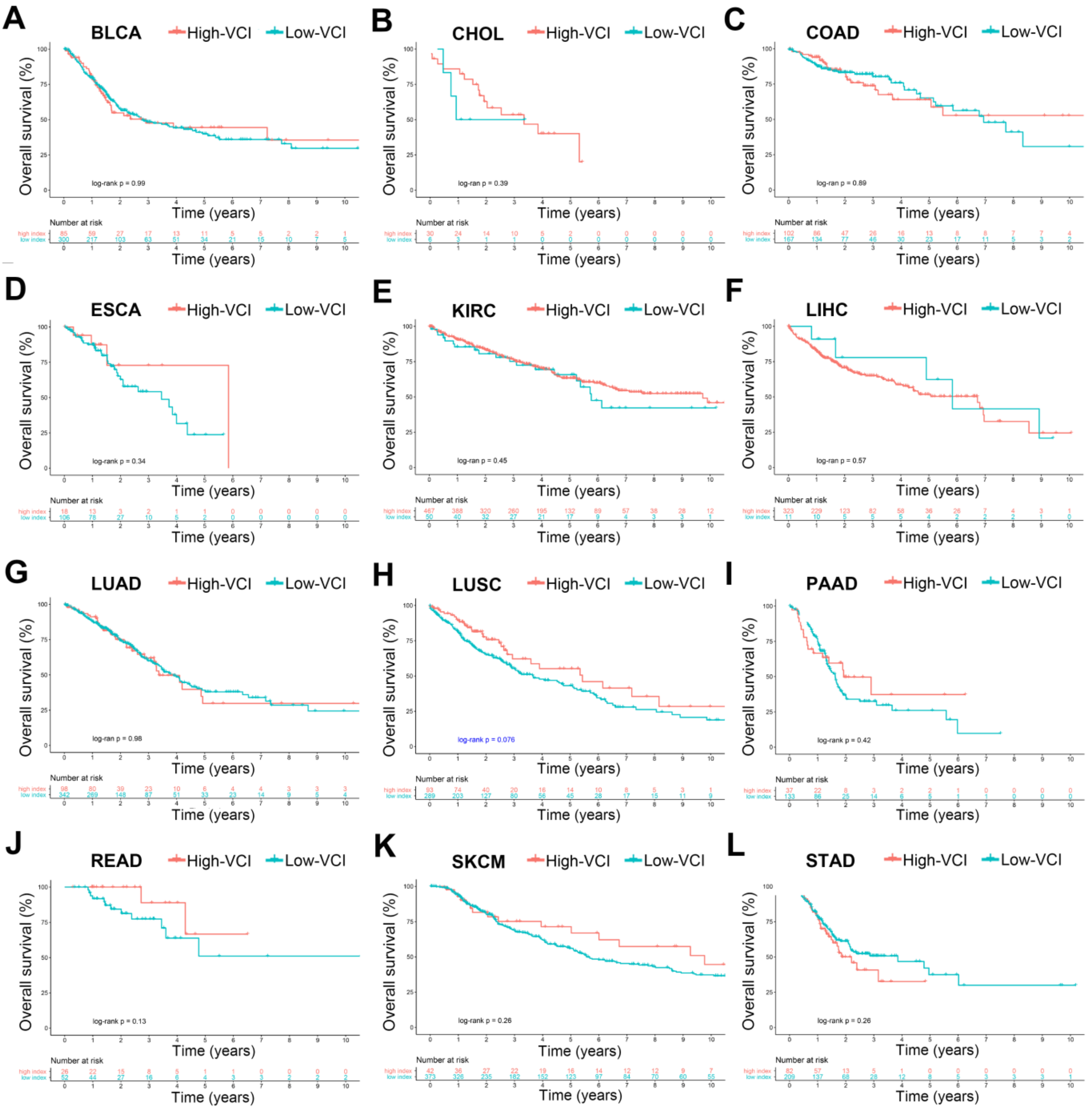

Figure S2. Relationship between vitamin C index (VCI) and overall survival (OS) in cancer patients. Kaplan–Meier analysis of patient survival based on their VCI in (**A**) bladder urothelial carcinoma (BLCA), (**B**) cholangiocarcinoma (CHOL), (**C**) colon adenocarcinoma (COAD), (**D**) esophageal carcinoma (ESCA), (**E**) kidney renal clear cell carcinoma (KIRC), (**F**) liver hepatocellular carcinoma (LIHC), (**G**) lung adenocarcinoma (LUAD), (**H**) lung squamous cell carcinoma (LUSC), (**I**) pancreatic adenocarcinoma (PAAD), (**J**) rectum adenocarcinoma (READ), (**K**) skin cutaneous melanoma (SKCM), and (**L**) stomach adenocarcinoma (STAD).

Supplementary Figure S3

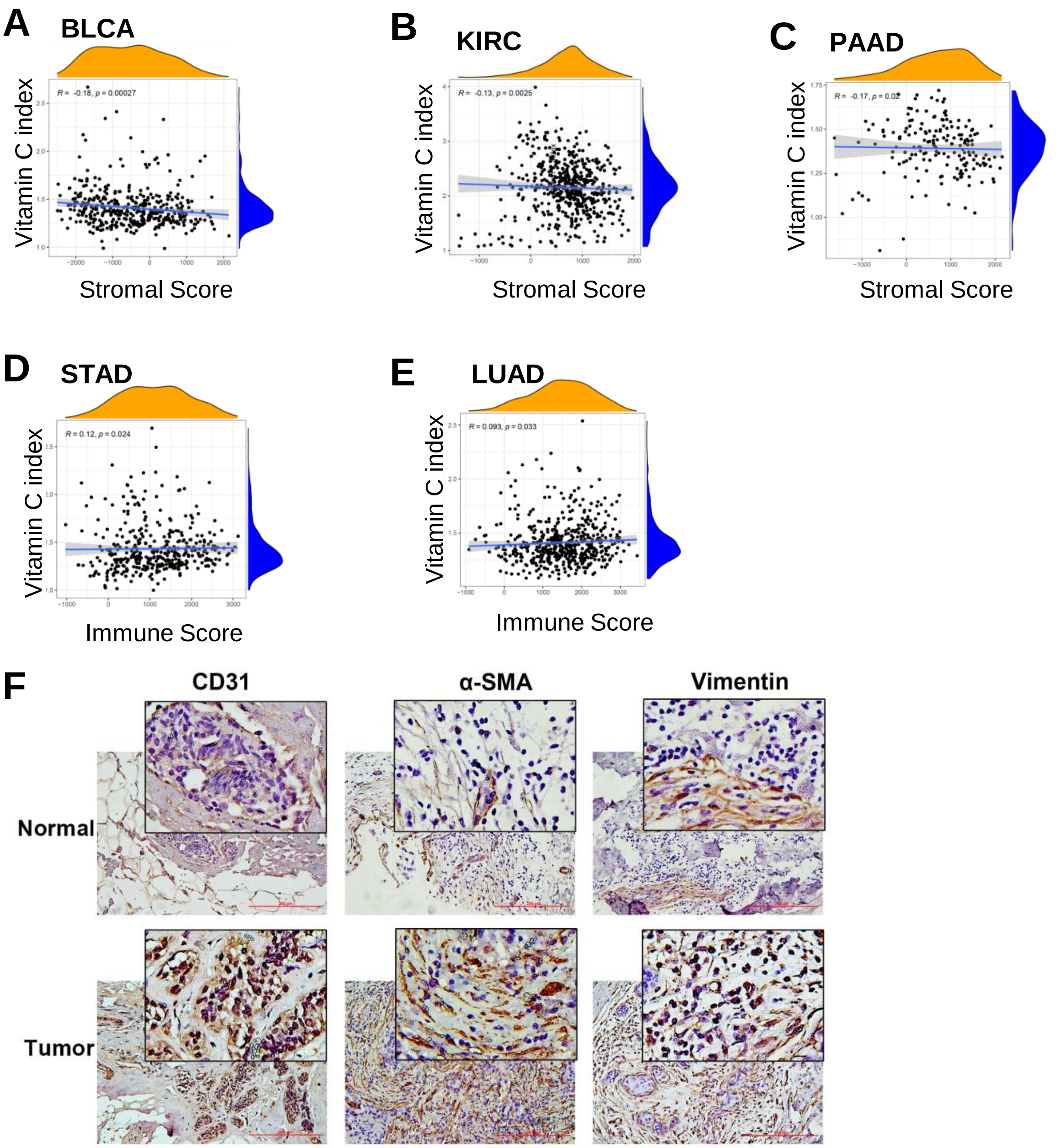

Figure S3. Correlation between Vitamin C index (VCI) and the tumor immune microenvironment (TME). The relationship between VCI and TME were evaluated by calculating stromal scores and immune scores based on gene expression characteristics of stromal and immune cells using the ESTIMATE prediction software. (**A-C**) Correlation between VCI and stromal score in bladder urothelial carcinoma (BLCA), kidney renal clear cell carcinoma (KIRA) and pancreatic adenocarcinoma (PAAD) as indicated. (**D-E**) Correlation between VCI and immune score in stomach adenocarcinoma (STAD) and lung adenocarcinoma (LUAD) as indicated. (**F**) The expression of fibroblast markers VIMENTIN, α-SMA and endothelial cell marker CD31 in clinical breast cancer tissues was detected by immunohistochemistry.

Supplementary Figure S4

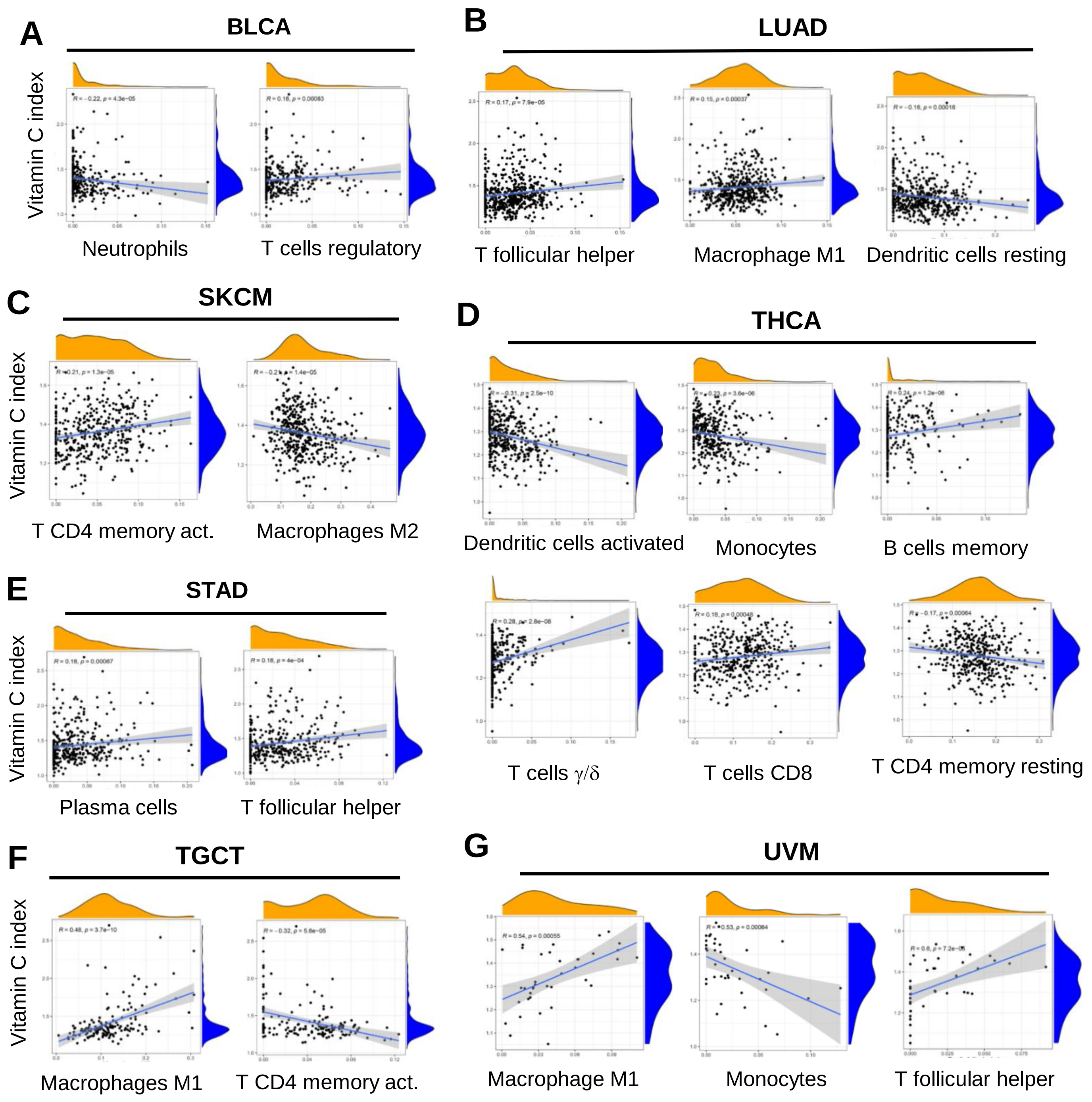

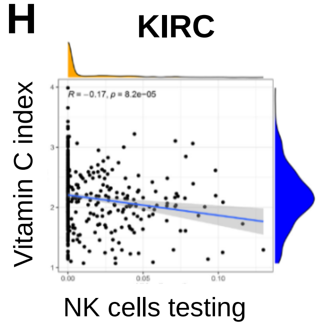

Figure S4. Association between vitamin C index (VCI) and infiltration of immune cells. The correlation between VCI and infiltration of immune cells in (**A**) bladder urothelial carcinoma (BLCA), (**B**) lung adenocarcinoma (LUAD), (**C**) skin cutaneous melanoma (SKCM), (**D**) thyroid carcinoma (THCA), (**E**) stomach adenocarcinoma (STAD), (**F**) testicular germ cell tumors (TGCT), (**G**) uveal melanoma (UVM), and (H) kidney renal clear cell carcinoma (KIRC). The specific types of infiltrated immune cells are indicated for each panel.

Supplementary Figure S5

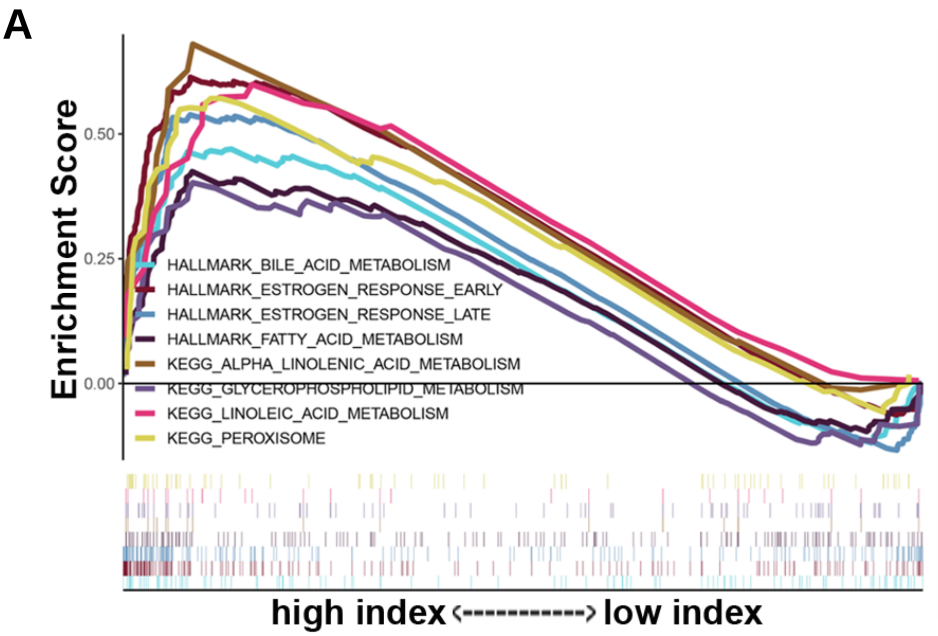

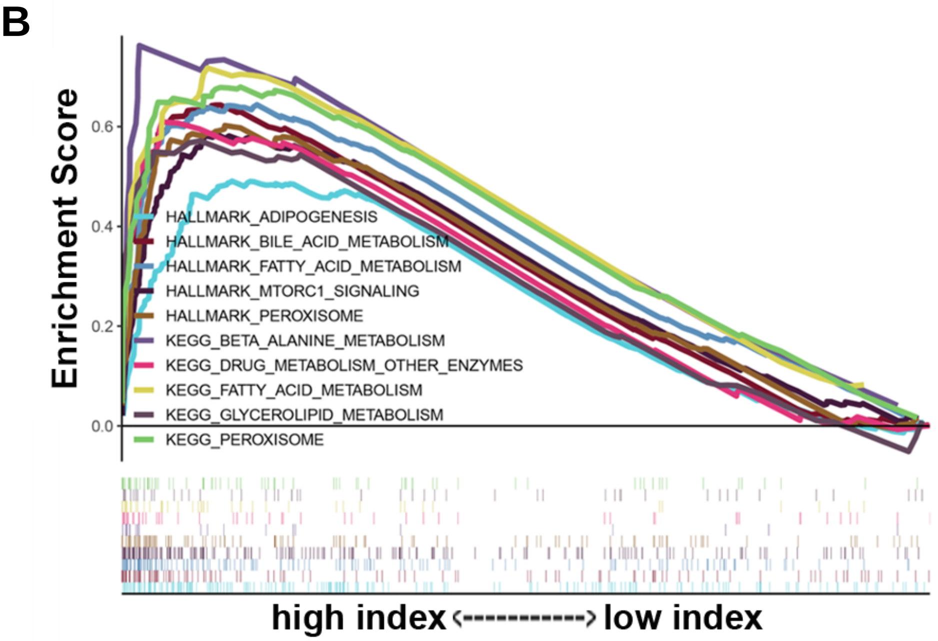

Figure S5. Enrichment of pathway analyses in cancers with high or low vitamin C index. The potential impact of vitamin C on molecular pathways was evaluated by gene set enrichment analysis in (**A**) breast cancer and (**B**) kidney renal papillary cell carcinoma.

Supplementary Figure S6

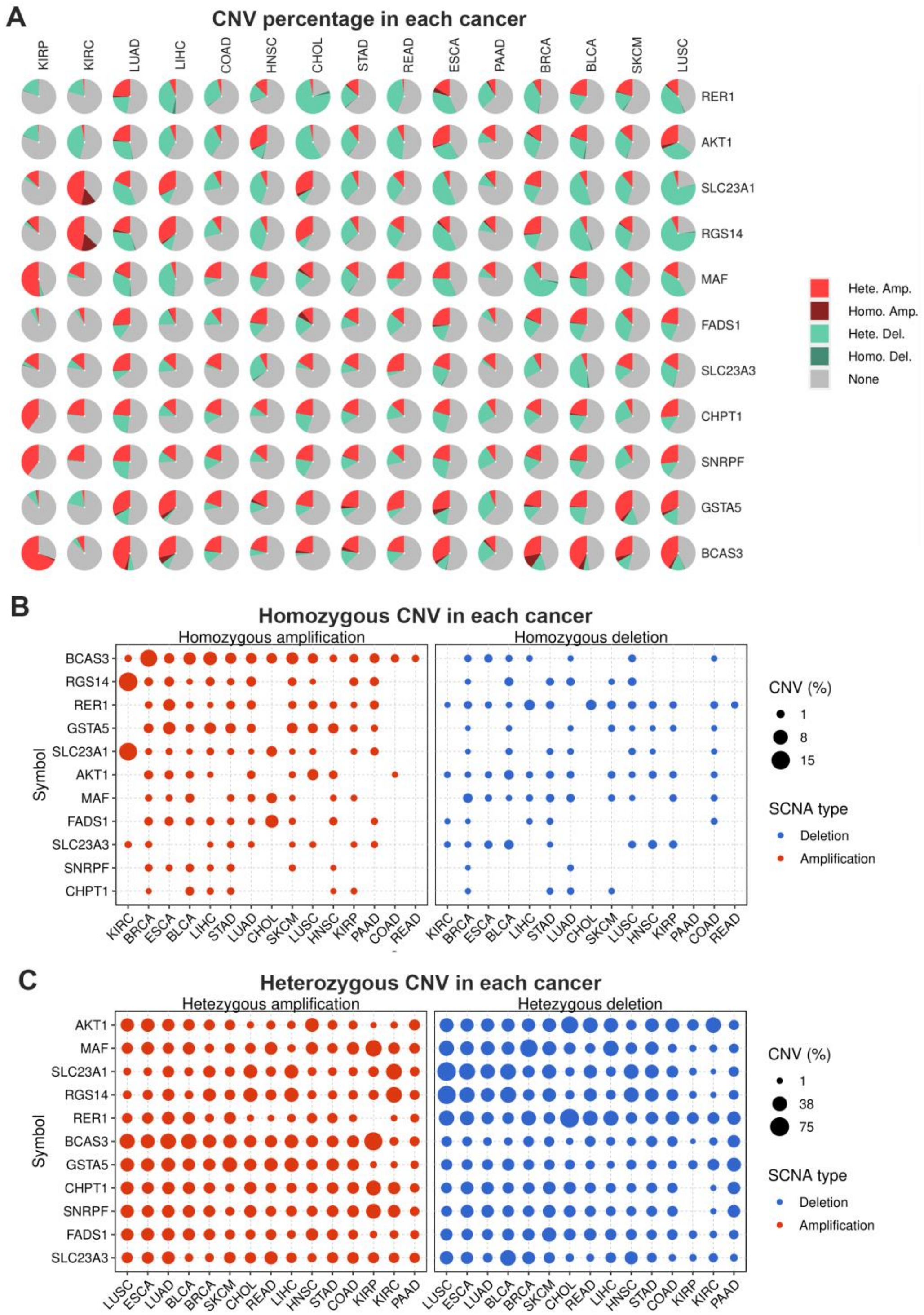

Figure S6. Variation of Vitamin C gene family in pan-cancer. (**A**) Alterations of CNV of EFNAs in cancers were assessed from GSCA. (**B**) Homozygous CNV in each cancer were assessed from GSCA. (**C**) Heterozygous CNV in each cancer were assessed from GSCA.
