# Supplementary material for: Genetically predicted vitamin C levels significantly affect patient survival and immunotypes in multiple cancer types
Source: Front Immunol. 2023 May 22;14:1177580. doi: 10.3389/fimmu.2023.1177580 (PMC10239825; doi:10.3389/fimmu.2023.1177580)
Supplement: Supplementary file 2 [file Table_1.docx]

**Table S1. Baseline characteristics of patients across 20 cancer types used in this study**

| **Characteristic** | **All**  **(N = 5,769)** | **BLCA**  **(N = 385)** | **BRCA**  **(N = 973)** | **CHOL**  **(N = 36)** | **COAD**  **(N = 269)** | **ESCA**  **(N = 124)** | **HNSC**  **(N = 419)** | **KICH**  **(N = 62)** | **KIRC**  **(N = 517)** | **KIRP**  **(N = 245)** | **LIHC**  **(N = 334)** | **LUAD**  **(N = 440)** | **LUSC**  **(N = 382)** | **MESO**  **(N = 84)** | **PAAD**  **(N = 170)** | **READ**  **(N = 78)** | **SKCM**  **(N = 415)** | **STAD**  **(N = 291)** | **TGCT**  **(N = 78)** | **THCA**  **(N = 413)** | **UVM**  **(N = 54)** |
| --- | --- | --- | --- | --- | --- | --- | --- | --- | --- | --- | --- | --- | --- | --- | --- | --- | --- | --- | --- | --- | --- |
| Age  median (IQR) yr | 61  (51–71) | 68  (60–76) | 58  (48–66) | 67  (57–72) | 65  (55–74) | 60  (53-71) | 61  (53–69) | 51  (43–62) | 60  (51–69) | 62  (54–71) | 61  (51-68) | 66  (59–73) | 68  (61–73) | 64  (57–69) | 65  (57–73) | 60  (53–71) | 58  (48–71) | 67  (58–72) | 32  (26–38) | 46  (35–58) | 60  (50–75) |
| Gender No. (%) |  |  |  |  |  |  |  |  |  |  |  |  |  |  |  |  |  |  |  |  |  |
| Male | 2,973 (52) | 287 (75) | 11 (1) | 16 (44) | 139 (52) | 104 (84) | 303 (72) | 37 (60) | 336 (65) | 177 (72) | 227 (68) | 195 (44) | 272 (71) | 69 (82) | 94 (55) | 45 (58) | 260 (63) | 184 (63) | 78 (100) | 110 (27) | 29 (54) |
| Female | 2,796 (48) | 98 (25) | 962 (99) | 20 (56) | 130 (48) | 20 (16) | 116 (28) | 25 (40) | 181 (35) | 68 (28) | 107 (32) | 245 (56) | 110 (29) | 15 (18) | 76 (45) | 33 (42) | 155 (37) | 107 (37) | 0 (0) | 303 (73) | 25 (46) |
| OS  median (IQR) yr | 2.0  (1.1–3.9) | 1.5  (0.9–2.5) | 2.4  (1.3–4.8) | 1.8  (1.0–3.3) | 1.8  (1.1–3.1) | 1.2  (0.9-1.9) | 1.8  (1.0–3.3) | 7.1  (2.8–8.5) | 3.3  (1.5–5.3) | 2.0  (1.1–3.6) | 1.6  (0.9-3.1) | 1.8  (1.2–3.0) | 1.7  (0.9–3.1) | 1.5  (0.8–2.3) | 1.3  (0.7–1.8) | 2.1  (1.3–3.2) | 2.9  (1.3–6.4) | 1.3  (0.8–2.1) | 3.1  (1.9–7.7) | 2.7  (1.6–4.4) | 2.3  (1.6–3.3) |

Abbreviations: BLCA, bladder urothelial carcinoma; BRCA, breast cancer; CHOL, cholangiocarcinoma; COAD, colon and rectal adenocarcinoma; ESCA, esophageal carcinoma; HNSC, head and neck squamous cell carcinoma; IQR, interquartile range; KICH, kidney chromophobe; KIRC, kidney clear cell carcinoma; KIRP, kidney renal papillary cell carcinoma; LIHC, liver hepatocellular carcinoma; LUAD, lung adenocarcinoma; LUSC, lung squamous cell carcinoma; MESO, mesothelioma; OS, overall survival; PAAD, pancreatic adenocarcinoma; READ, rectum adenocarcinoma; SKCM, skin cutaneous melanoma; STAD, stomach adenocarcinoma; TCGA, The Cancer Genome Atlas; TGCT, testicular germ cell tumors; THCA, thyroid carcinoma; UVM, uveal melanoma.
