# Supplementary material for: Genetically predicted vitamin C levels significantly affect patient survival and immunotypes in multiple cancer types
Source: Front Immunol. 2023 May 22;14:1177580. doi: 10.3389/fimmu.2023.1177580 (PMC10239825; doi:10.3389/fimmu.2023.1177580)
Supplement: Supplementary file 3 [file Table_2.docx]

**Table S2. Comparison of clinicopathological features between patients with high-VCI and low-VCI in multiple cancer types.**

| **Cancer Types** | **VCI** | **Age** | |  | **Gender** | |  | **Race** | | |  | **Disease stage** | |  |
| --- | --- | --- | --- | --- | --- | --- | --- | --- | --- | --- | --- | --- | --- | --- |
|  |  | **Median (IQR)** | | ***P^*^*** | **Female** | **Male** | ***P***^†^ | **White** | **Asian** | **Others** | ***P***^†^ | **I-II** | **III-IV** | ***P***^†^ |
| **ALL** | Low | 63 (53–71) |  | **0.01** | 1881 (50.1) | 1870 (49.9) | **0.01** | 3148 (83.9) | 264 (7.0) | 339 (9.0) | **0.01** | 2249 (60.0) | 1502 (40.0) | **0.01** |
|  | High | 60 (50–70) |  |  | 915 (45.3) | 1103 (54.7) |  | 1553 (77.0) | 234 (11.6) | 231 (11.4) |  | 1347 (66.7) | 671 (33.3) |  |
| **BLCA** | Low | 70 (62–78) |  | 0.07 | 79 (26.3) | 221 (73.7) | 0.55 | 245 (81.7) | 37 (12.3) | 18 (6.0) | 0.39 | 96 (32.0) | 204 (68.0) | 0.81 |
|  | High | 68 (60–76) |  |  | 19 (22.4) | 66 (77.6) |  | 75 (88.2) | 6 (7.1) | 4 (4.7) |  | 29 (34.1) | 56 (65.9) |  |
| **BRCA** | Low | 60 (50–68) |  | **0.01** | 621 (98.9) | 7 (1.1) | 0.99 | 474 (75.5) | 38 (6.1) | 116 (18.5) | 0.85 | 484 (77.1) | 144 (22.9) | 0.19 |
|  | High | 56 (48–66) |  |  | 341 (98.8) | 4 (1.2) |  | 266 (77.1) | 20 (5.8) | 59 (17.1) |  | 252 (73.0) | 93 (27.0) |  |
| **CHOL** | Low | 70 (68–72) |  | 0.49 | 2 (33.3) | 4 (66.7) | 0.37 | 4 (66.7) | 1 (16.7) | 1 (16.7) | 0.19 | 3 (50.0) | 3 (50.0) | 0.11 |
|  | High | 63 (56–72) |  |  | 18 (60.0) | 12 (40.0) |  | 27 (90.0) | 2 (6.7) | 1 (3.3) |  | 25 (83.3) | 5 (16.7) |  |
| **COAD** | Low | 67 (56–75) |  | 0.12 | 78 (46.7) | 89 (53.3) | 0.58 | 124 (74.3) | 8 (4.8) | 35 (21.0) | 0.76 | 91 (54.5) | 76 (45.5) | 0.66 |
|  | High | 63 (53–73) |  |  | 52 (51.0) | 50 (49.0) |  | 76 (74.5) | 3 (2.9) | 23 (22.5) |  | 52 (51.0) | 50 (49.0) |  |
| **ESCA** | Low | 60 (53–71) |  | 0.79 | 16 (15.1) | 90 (84.9) | 0.49 | 72 (67.9) | 32 (30.2) | 2 (1.9) | 0.48 | 69 (65.1) | 37 (34.9) | 0.34 |
|  | High | 63 (51–73) |  |  | 4 (22.2) | 14 (77.8) |  | 11 (61.1) | 6 (33.3) | 1 (5.6) |  | 9 (50.0) | 9 (50.0) |  |
| **HNSC** | Low | 61 (54–69) |  | **0.01** | 113 (29.4) | 272 (70.6) | **0.02** | 340 (88.3) | 10 (2.6) | 35 (9.1) | 0.20 | 85 (22.1) | 300 (77.9) | 0.23 |
|  | High | 57 (50–61) |  |  | 3 (8.8) | 31 (91.2) |  | 28 (82.4) | 0 (0.0) | 6 (17.6) |  | 4 (11.8) | 30 (88.2) |  |
| **KIRC** | Low | 63 (55–70) |  | 0.35 | 10 (20.0) | 40 (80.0) | **0.03** | 41 (82.0) | 0 (0.0) | 9 (18.0) | 0.15 | 33 (66.0) | 17 (34.0) | 0.54 |
|  | High | 60 (51–69) |  |  | 171 (36.6) | 296 (63.4) |  | 414 (88.0) | 8 (1.7) | 45 (9.6) |  | 282 (60.4) | 185 (39.6) |  |
| **KIRP** | Low | 65 (55–75) |  | 0.79 | 8 (44.4) | 10 (55.6) | 0.11 | 10 (55.6) | 1 (5.6) | 7 (38.9) | 0.12 | 13 (72.2) | 5 (27.8) | 0.78 |
|  | High | 62 (54–70) |  |  | 60 (26.4) | 167 (73.6) |  | 171 (75.3) | 5 (2.2) | 51 (22.5) |  | 170 (74.9) | 57 (25.1) |  |
| **LIHC** | Low | 56 (50–72) |  | 0.72 | 6 (54.5) | 5 (45.5) | 0.11 | 8 (72.7) | 3 (27.3) | 0 (0.0) | 0.32 | 6 (54.5) | 5 (45.5) | 0.16 |
|  | High | 61 (51–68) |  |  | 101 (31.3) | 222 (68.7) |  | 157 (48.6) | 151 (46.7) | 15 (4.6) |  | 243 (75.2) | 80 (24.8) |  |
| **LUAD** | Low | 65 (59–72) |  | 0.21 | 191 (55.8) | 151 (44.2) | 0.99 | 296 (86.5) | 5 (1.5) | 41 (12.0) | 0.82 | 269 (78.7) | 73 (21.3) | 0.47 |
|  | High | 69 (60–74) |  |  | 54 (55.1) | 44 (44.9) |  | 85 (86.7) | 2 (2.0) | 11 (11.2) |  | 81 (82.7) | 17 (17.3) |  |
| **LUSC** | Low | 68 (61–73) |  | 0.97 | 87 (30.1) | 202 (69.9) | 0.39 | 257 (88.9) | 6 (2.1) | 26 (9.0) | 0.13 | 240 (83.0) | 49 (17.0) | 0.44 |
|  | High | 68 (61–74) |  |  | 23 (24.7) | 70 (75.3) |  | 87 (93.5) | 3 (3.2) | 3 (3.2) |  | 81 (87.1) | 12 (12.9) |  |
| **PAAD** | Low | 65 (58–74) |  | 0.43 | 60 (45.1) | 73 (54.9) | 0.99 | 118 (88.7) | 10 (7.5) | 5 (3.8) | 0.71 | 125 (94.0) | 8 (6.0) | 0.20 |
|  | High | 64 (56–71) |  |  | 16 (43.2) | 21 (56.8) |  | 35 (94.6) | 1 (2.7) | 1 (2.7) |  | 37 (100.0) | 0 (0.0) |  |
| **READ** | Low | 62 (53–70) |  | 0.84 | 22 (42.3) | 30 (57.7) | 0.99 | 47 (90.4) | 1 (1.9) | 4 (7.7) | 0.99 | 19 (36.5) | 33 (63.5) | 0.13 |
|  | High | 58 (53–72) |  |  | 11 (42.3) | 15 (57.7) |  | 24 (92.3) | 0 (0.0) | 2 (7.7) |  | 15 (57.7) | 11 (42.3) |  |
| **SKCM** | Low | 58 (48–70) |  | 0.90 | 138 (37.0) | 235 (63.0) | 0.78 | 362 (97.1) | 11 (3.0) | 0 (0.0) | 0.12 | 201 (53.9) | 172 (46.1) | 0.81 |
|  | High | 59 (45–73) |  |  | 17 (40.5) | 25 (59.5) |  | 40 (95.2) | 1 (2.4) | 1 (2.4) |  | 24 (57.1) | 18 (42.9) |  |
| **STAD** | Low | 67 (58–72) |  | 0.58 | 84 (40.2) | 125 (59.8) | 0.07 | 160 (76.6) | 45 (21.5) | 4 (1.9) | **0.01** | 100 (47.8) | 109 (52.2) | 0.92 |
|  | High | 67 (58–72) |  |  | 23 (28.0) | 59 (72.0) |  | 49 (59.8) | 26 (31.7) | 7 (8.5) |  | 38 (46.3) | 44 (53.7) |  |
| **TGCT** | Low | 32 (26–39) |  | 0.25 | -- | 72 (100.0) | -- | 65 (90.3) | 2 (2.8) | 5 (6.9) | 0.49 | 62 (86.1) | 10 (13.9) | 0.06 |
|  | High | 32 (28–33) |  |  | -- | 6 (100.0) |  | 5 (83.3) | 0 (0.0) | 1 (16.7) |  | 3 (50.0) | 3 (50.0) |  |

Notes:

(1) * *P*-values were calculated Mann-Whitney U test; † *P*-values were calculated by Chi-square test.

(2) Abbreviations: VCI, vitamin C index; BLCA, bladder urothelial carcinoma; BRCA, breast cancer; CHOL, cholangiocarcinoma; COAD, colon and rectal adenocarcinoma; ESCA, esophageal carcinoma; HNSC, head and neck squamous cell carcinoma; IQR, interquartile range; KICH, kidney chromophobe; KIRC, kidney clear cell carcinoma; KIRP, kidney renal papillary cell carcinoma; LIHC, liver hepatocellular carcinoma; LUAD, lung adenocarcinoma; LUSC, lung squamous cell carcinoma; MESO, mesothelioma; PAAD, pancreatic adenocarcinoma; READ, rectum adenocarcinoma; SKCM, skin cutaneous melanoma; STAD, stomach adenocarcinoma; TCGA, The Cancer Genome Atlas; TGCT, testicular germ cell tumors; THCA, thyroid carcinoma; UVM, uveal melanoma.

(3) Since KICH patients were all in the low-VCI group whereas MESO, THCA and UVM patients were all in the high-VCI group, these 4 cancer types could not be included in the comparison analysis.
