# Supplementary material for: Genetically predicted vitamin C levels significantly affect patient survival and immunotypes in multiple cancer types
Source: Front Immunol. 2023 May 22;14:1177580. doi: 10.3389/fimmu.2023.1177580 (PMC10239825; doi:10.3389/fimmu.2023.1177580)
Supplement: Supplementary file 4 [file Table_3.docx]

**Table S3. Cox proportional hazards analyses of clinical covariates in various cancer types for overall survival**

| **Cancer types** | **Covariates** | **AHR** | **95%CI** | **P-values^†^** |
| --- | --- | --- | --- | --- |
| **All** | VCindex | 0.87 | 0.78–0.98 | 0.02 |
|  | Age (continuous) | 1.03 | 1.03–1.04 | <0.01 |
|  | Gender (Male *vs.* Female) | 1.35 | 1.23–1.48 | <0.01 |
|  | Race |  |  | <0.01 |
|  | Asian *vs.* White (reference) | 1.32 | 1.09–1.61 |  |
|  | Black *vs.* White (reference) | 1.03 | 0.87–1.21 |  |
|  | Stage (III-IV *vs.* I-II) | 2.12 | 1.93–2.33 | <0.01 |
| **BLCA** | VCindex | 1.04 | 0.44–2.49 | 0.92 |
|  | Age (continuous) | 1.03 | 1.02–1.05 | <0.01 |
|  | Gender (Male *vs.* Female) | 0.83 | 0.59–1.16 | 0.28 |
|  | Race |  |  | 0.66 |
|  | Asian *vs.* White (reference) | 0.86 | 0.43–1.70 |  |
|  | Black *vs.* White (reference) | 1.49 | 0.84–2.63 |  |
|  | Stage (III-IV *vs.* I-II) | 2.09 | 1.42–3.08 | <0.01 |
| **BRCA** | VCindex | 0.14 | 0.05–0.40 | <0.01 |
|  | Age (continuous) | 1.04 | 1.02–1.05 | <0.01 |
|  | Gender (Male *vs.* Female) | 0.55 | 0.08–3.98 | 0.55 |
|  | Race |  |  | 0.89 |
|  | Asian *vs.* White (reference) | 1.08 | 0.34–3.44 |  |
|  | Black *vs.* White (reference) | 1.18 | 0.77–1.81 |  |
|  | Stage (III-IV *vs.* I-II) | 2.77 | 1.97–3.91 | <0.01 |
| **CHOL** | VCindex | 1.15 | 0.30–4.36 | 0.84 |
|  | Age (continuous) | 1.01 | 0.97–1.06 | 0.58 |
|  | Gender (Male *vs.* Female) | 1.31 | 0.44–3.94 | 0.63 |
|  | Race |  |  | 0.22 |
|  | Asian *vs.* White (reference) | 2.94 | 0.52–16.64 |  |
|  | Black *vs.* White (reference) | 2.14 | 0.24–19.25 |  |
|  | Stage (III-IV *vs.* I-II) | 2.00 | 0.55–7.28 | 0.29 |
| **COAD** | VCindex | 1.68 | 0.39–7.13 | 0.48 |
|  | Age (continuous) | 1.03 | 1.01–1.05 | 0.01 |
|  | Gender (Male *vs.* Female) | 1.26 | 0.74–2.15 | 0.40 |
|  | Race |  |  | 0.46 |
|  | Asian *vs.* White (reference) | 1.74 | 0.40–7.46 |  |
|  | Black *vs.* White (reference) | 1.13 | 0.59–2.17 |  |
|  | Stage (III-IV *vs.* I-II) | 2.72 | 1.58–4.67 | <0.01 |
| **ESCA** | VCindex | 1.27 | 0.15–10.56 | 0.83 |
|  | Age (continuous) | 0.99 | 0.96–1.02 | 0.61 |
|  | Gender (Male *vs.* Female) | 1.85 | 0.53–6.42 | 0.34 |
|  | Race |  |  | 0.96 |
|  | Asian *vs.* White (reference) | 1.02 | 0.39–2.66 |  |
|  | Black *vs.* White (reference) | 1.70 | 0.21–13.90 |  |
|  | Stage (III-IV *vs.* I-II) | 2.71 | 1.29–5.69 | <0.01 |
| **HNSC** | VCindex | 0.20 | 0.07–0.59 | <0.01 |
|  | Age (continuous) | 1.02 | 1.00–1.03 | 0.01 |
|  | Gender (Male *vs.* Female) | 0.80 | 0.58–1.12 | 0.19 |
|  | Race |  |  | 0.50 |
|  | Asian *vs.* White (reference) | 1.41 | 0.52–3.83 |  |
|  | Black *vs.* White (reference) | 1.36 | 0.83–2.24 |  |
|  | Stage (III-IV *vs.* I-II) | 1.98 | 1.30–3.00 | <0.01 |
| **KIRC** | VCindex | 0.66 | 0.48–0.92 | 0.01 |
|  | Age (continuous) | 1.03 | 1.02–1.05 | <0.01 |
|  | Gender (Male *vs.* Female) | 0.95 | 0.69–1.33 | 0.78 |
|  | Race |  |  | 0.49 |
|  | Asian *vs.* White (reference) | 0.50 | 0.07–3.60 |  |
|  | Black *vs.* White (reference) | 0.90 | 0.49–1.67 |  |
|  | Stage (III-IV *vs.* I-II) | 4.10 | 2.96–5.68 | <0.01 |
| **KIRP** | VCindex | 0.57 | 0.26–1.25 | 0.16 |
|  | Age (continuous) | 1.01 | 0.97–1.03 | 0.93 |
|  | Gender (Male *vs.* Female) | 0.74 | 0.37–1.51 | 0.41 |
|  | Race |  |  | 0.09 |
|  | Asian *vs.* White (reference) | 6.61 | 0.74–59.25 |  |
|  | Black *vs.* White (reference) | 0.99 | 0.43–2.29 |  |
|  | Stage (III-IV *vs.* I-II) | 6.52 | 3.29–12.91 | <0.01 |
| **LIHC** | VCindex | 1.31 | 0.83–2.06 | 0.25 |
|  | Age (continuous) | 1.01 | 1.00–1.03 | 0.10 |
|  | Gender (Male *vs.* Female) | 0.80 | 0.53–1.21 | 0.29 |
|  | Race |  |  | 0.71 |
|  | Asian *vs.* White (reference) | 1.09 | 0.70–1.67 |  |
|  | Black *vs.* White (reference) | 2.51 | 1.04–6.07 |  |
|  | Stage (III-IV *vs.* I-II) | 2.54 | 1.72–3.74 | <0.01 |
| **LUAD** | VCindex | 1.58 | 0.68–3.65 | 0.29 |
|  | Age (continuous) | 1.00 | 0.99–1.02 | 0.55 |
|  | Gender (Male *vs.* Female) | 1.06 | 0.78–1.45 | 0.71 |
|  | Race |  |  | 0.42 |
|  | Asian *vs.* White (reference) | 0.44 | 0.06–3.18 |  |
|  | Black *vs.* White (reference) | 0.73 | 0.42–1.26 |  |
|  | Stage (III-IV *vs.* I-II) | 2.21 | 1.58–3.10 | <0.01 |
| **LUSC** | VCindex | 0.53 | 0.18–1.62 | 0.27 |
|  | Age (continuous) | 1.02 | 1.00–1.04 | 0.05 |
|  | Gender (Male *vs.* Female) | 1.60 | 1.11–2.32 | 0.01 |
|  | Race |  |  | 0.22 |
|  | Asian *vs.* White (reference) | 1.89 | 0.69–5.16 |  |
|  | Black *vs.* White (reference) | 1.66 | 1.02–2.72 |  |
|  | Stage (III-IV *vs.* I-II) | 1.73 | 1.18–2.53 | <0.01 |
| **PAAD** | VCindex | 2.50 | 0.62–10.00 | 0.20 |
|  | Age (continuous) | 1.03 | 1.00–1.05 | 0.02 |
|  | Gender (Male *vs.* Female) | 0.78 | 0.51–1.20 | 0.26 |
|  | Race |  |  | 0.74 |
|  | Asian *vs.* White (reference) | 0.85 | 0.34–2.15 |  |
|  | Black *vs.* White (reference) | 1.02 | 0.37–2.83 |  |
|  | Stage (III-IV *vs.* I-II) | 0.67 | 0.21–2.16 | 0.51 |
| **READ** | VCindex | 0.01 | 0.001–0.38 | 0.02 |
|  | Age (continuous) | 1.14 | 1.04–1.26 | <0.01 |
|  | Gender (Male *vs.* Female) | 1.16 | 0.32–46.01 | 0.80 |
|  | Race |  |  | 0.99 |
|  | Asian *vs.* White (reference) | -- | -- | -- |
|  | Black *vs.* White (reference) | -- | -- | -- |
|  | Stage (III-IV *vs.* I-II) | 0.67 | 0.21–2.16 | 0.23 |
| **SKCM** | VCindex | 0.98 | 0.28–3.39 | 0.97 |
|  | Age (continuous) | 1.02 | 1.01–1.03 | <0.01 |
|  | Gender (Male *vs.* Female) | 0.96 | 0.72–1.30 | 0.81 |
|  | Race |  |  | <0.01 |
|  | Asian *vs.* White (reference) | 3.69 | 1.69–8.06 |  |
|  | Black *vs.* White (reference) | 0.67 | 0.09–5.05 |  |
|  | Stage (III-IV *vs.* I-II) | 1.68 | 1.26–2.25 | <0.01 |
| **STAD** | VCindex | 1.68 | 0.81–3.50 | 0.17 |
|  | Age (continuous) | 1.02 | 1.00–1.04 | 0.05 |
|  | Gender (Male *vs.* Female) | 1.17 | 0.78–1.74 | 0.45 |
|  | Race |  |  | 0.34 |
|  | Asian *vs.* White (reference) | 0.78 | 0.47–1.30 |  |
|  | Black *vs.* White (reference) | 1.22 | 0.58–2.58 |  |
|  | Stage (III-IV *vs.* I-II) | 1.64 | 1.12–2.42 | 0.01 |

Abbreviations: AHR, adjusted hazard ratio; BLCA, bladder urothelial carcinoma; BRCA, breast cancer; CESC, cervical cancer; COAD, colon and rectal adenocarcinoma; CI, confidence interval; HNSC, head and neck squamous cell carcinoma; IQR, interquartile range; KIRC, kidney clear cell carcinoma; LIHC, liver hepatocellular carcinoma; LUAD, lung adenocarcinoma; LUSC, lung squamous cell carcinoma; PAAD, pancreatic adenocarcinoma; PRAD, prostate adenocarcinoma; SKCM, skin cutaneous melanoma; STAD, stomach adenocarcinoma; THCA, thyroid carcinoma.

* KICH was excluded from the analyses due to its samples were all in low vitamin C index group; MESO, THCA and UVM were excluded from the analyses due to its samples were all in high vitamin C index group. TGCT were excluded from the multivariable analyses due to the limited number of events.

† age, sex, race, stage were included in the Cox regression model
